# Ultrathin ALD Coatings of Zr and V Oxides on Anodic TiO_2_ Nanotube Layers: Comparison of the Osteoblast Cell Growth

**DOI:** 10.1021/acsami.4c19142

**Published:** 2024-12-28

**Authors:** Kaushik Baishya, Jana Bacova, Bachar Al Chimali, Jan Capek, Jan Michalicka, Gael Gautier, Brice Le Borgne, Tomas Rousar, Jan M. Macak

**Affiliations:** † Central European Institute of Technology, Brno University of Technology, Purkynova 123, 612 00 Brno, Czech Republic; ‡ Department of Biological and Biochemical Sciences, Faculty of Chemical Technology, University of Pardubice, Studentska 573, 532 10 Pardubice, Czech Republic; § GREMAN UMR-CNRS 7347, INSA Centre Val de Loire, Université de Tours, 37071 Tours Cedex 2, France; ∥ Center of Materials and Nanotechnologies, Faculty of Chemical Technology, University of Pardubice, Nam. Cs. Legii, 532 10 Pardubice, Czech Republic

**Keywords:** TiO_2_ nanotube layers, ZrO_2_, V_2_O_5_, atomic layer deposition, MG-63, cell viability.

## Abstract

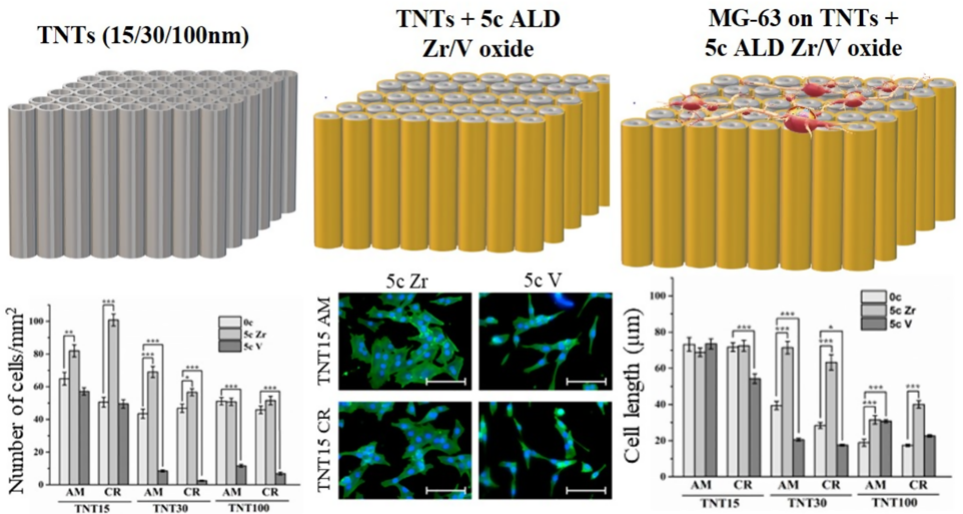

The current study investigates and compares the biological effects of ultrathin conformal coatings of zirconium dioxide (ZrO_2_) and vanadium pentoxide (V_2_O_5_) on osteoblastic MG-63 cells grown on TiO_2_ nanotube layers (TNTs). Coatings were achieved by the atomic layer deposition (ALD) technique. TNTs with average tube diameters of 15, 30, and 100 nm were fabricated on Ti substrates (via electrochemical anodization) and were used as primary substrates for the study. The MG-63 cell growth and proliferation after 48 h of incubation on hybrid TNTs/ZrO_2_ and TNTs/V_2_O_5_ surfaces was evaluated in comparison to the uncoated TNTs of each diameter. The density of viable MG-63 cells was assessed for all the TNT surfaces, along with the cell morphology and the spreading behavior (i.e., the cell length). The ultrathin coatings retained the original morphology of the TNTs but changed the surface chemical composition, wettability, and cell behavior, whose interplay is the subject of the present investigation. These findings offer interesting views on the influence of the composition of biomedical implant surfaces, triggered by ALD ultrathin coatings on them. The outcomes of this work shed light on the assessment of the biocompatibility of the two different ALD coatings.

## 1 Introduction

Over the last decades, titanium dioxide (TiO_2_), in the form of nanotubes or thin films, has been extensively studied for its bioinertness and good biocompatibility,^1−4^ being also at the same time an uppermost layer of Ti-based biomedical implants. Anodization of Ti under optimized conditions yields TNTs (grown on the Ti surface) with remarkable biocompatibility, exceptional stability, adjustable dimensions, and favorable cell viability. Moreover, the interaction of endothelial cells^5^ and mesenchymal stem cells (MSCs)^6^ with TNTs is known to largely depend on the nanotube diameter. In recent studies, a significantly improved cell response was demonstrated for WI-38, MG-63, SH-SY5Y,^7,8^ human gingival fibroblast (hGFs),^9^ and A549 and SH-SY5Y cells^10^ achieved by ALD ultrathin TiO_2_ coatings on TNTs grown on Ti^7−10^ and Ti alloys.^11^ In addition, the use of a thin TiO_2_ coating was shown to preserve the original amorphous nature of the TNTs,^12^ but to chemically change the uppermost surface and subsequently increase the cellular adhesion.^9^ The amorphous TiO_2_ surfaces have been reported to exhibit improved cellular activity of MSCs.^5^

Other metal oxide coatings for implant applications have been comparatively less explored but may be promising. Among these, V_2_O_5_ and ZrO_2_ embedded within TiO_2_ are naturally present on the surfaces of implant materials, depending on the composition, like the TiAlV alloy,^13^ Ti6Al4V,^14^ TiZr,^15^ Ti-Zr-6Al-4V,^16^ etc. However, the influence of the complete coating of these oxides over TiO_2_ surfaces of different kinds on the cellular response was not investigated in sufficient detail. However, understanding the influence of V_2_O_5_ and ZrO_2_ coatings on cellular interactions on TiO_2_ can be crucial for the performance of implant materials.

ZrO_2_ has been widely used in dental implants and as femoral heads in total hip replacement to resist corrosion and wear.^17,18^ Ti implants with ALD-coated ZrO_2_ can be used to reduce peri-implantitis by inhibiting the adhesion of oral bacteria, such as *S. mutans* and *P. gingivalis*.^19^ The role of vanadium oxides, however, has been controversial in the literature; some studies report its positive influence and some its negative influence. Low doses of different vanadium oxides have been shown to promote the proliferation of fibroblasts and osteoblast-like cells.^20−22^ On the other hand, studies revealed that, for vanadium compounds at higher concentrations in humans and animals, vanadium may exert various toxic effects.^23^ For example, vanadium released from microarc oxidized porous Ti6Al4V was shown to possess an adverse influence on the cell viability of human bone marrow-derived MSCs.^14^ In another study, a VO_2_ nano coating was exploited as an antitumor surface material for implantable biomaterials/devices for cancer treatments.^24^ Another study demonstrated that TiO_2_ coatings doped with vanadium can function as a vanadium delivery device, which can stimulate cell proliferation based on the doses of vanadium in the matrix.^25^ These metal–organic matrices can potentially control cell attachment, proliferation, extracellular matrix formation, and tissue growth from integrated biomaterial systems.

In the current study, the effect of ultrathin ALD coatings of ZrO_2_ and V_2_O_5_ on TNT surfaces on MG-63 cell growth is exploited and compared. The MG-63 cell line was used because the effect of surface ALD coating on cell adhesion, proliferation, and growth has been extensively studied using these particular MG-63 cells in many recent reports.^8,26,27^ Anodized TNT layers with three different nanotube diameters (approximately 15, 30, and 100 nm) were used for the study. Part of the TNTs was annealed in a muffle oven at 400 °C for 1 h to become crystalline. Part of amorphous and crystalline TNTs was coated with additional ZrO_2_ and V_2_O_5_ ultrathin coatings using ALD. The morphology and composition of all surfaces were investigated by scanning electron microscopy (SEM), X-ray photoelectron spectroscopy (XPS), atomic force microscopy (AFM), and water contact angle analysis (WCA). MG-63 cells were grown on amorphous or crystalline, coated, or uncoated TNTs for 48 h. The effect of all of the ALD-coated and uncoated surfaces on the cell growth and morphology was assessed and compared.

## 2 Experimental Section

### 2.1 Materials Synthesis and Characterization

Preceding all experimental investigations, TiO_2_ nanotube layers (TNTs) with three distinct inner diameters (approximately 15, 30, and 100 nm) were fabricated. For that Ti foils (99.7% purity, 0.127 mm thick) were acquired from Sigma-Aldrich and cut into 1.5 cm^2^ pieces. These pieces were then cleaned by sonicating in isopropanol, followed by acetone, and then in isopropanol again, after which they were left to air-dry at room temperature. The anodization setup consisted of an electrochemical cell with a circular opening that exposed 1 cm^2^ of the Ti foil to the electrolyte, along with a high-voltage potentiostat (ITECH, IT6512C, Taiwan) connected to a digital multimeter (Keithley 2100). A platinum foil served as the counter electrode. Anodization was performed at 4, 10, and 28 V for 3 h at room temperature, using a glycerol-based electrolyte composed of 50% deionized water and 0.27 M ammonium fluoride (NH_4_F) to TNT15 AM, TNT30 AM, and TNT100 AM, respectively. The resulting amorphous TNTs were then sonicated in 2-propanol and air-dried. Parts of the samples were annealed at 400 °C for 1 h in a static atmosphere of air in a laboratory muffle oven (with a sweep rate of 2.1 °C min^–1^) to obtain crystalline TNTs of diameters 15, 30, and 100 nm, further denoted as TNT15 CR, TNT30 CR and TNT100 CR, respectively). The XRD data confirming the change in the phase composition of the TNTs after annealing were shown in our previous study.^7^Table 1 provides an overview of all samples investigated in this work. Parts of the samples were further processed to obtain ultrathin coatings of ZrO_2_ and V_2_O_5_ by atomic layer deposition (ALD). The ALD processing was carried out using the commercial TFS200 tool (Beneq). ALD of ZrO_2_ was carried out at 275 °C using tris(dimethylamino)cyclopentadienyl zirconium, CpZr(NMe_2_)_3_, and ZyALD (Air Liquide) heated at 75 °C as the Zr precursor and ozone as the oxygen source. High-purity N_2_ (99.9999%) was the carrier and purging gas at a flow rate of 400 standard cubic centimeters per minute (sccm). Under these deposition conditions, one growth ALD ZrO_2_ cycle (1c) was defined by the following sequence: ZyALD pulse (300 ms)–N_2_ purge (20 s)–O_3_ pulse (2 s)–N_2_ purge (20 s). Parts of the amorphous and crystalline TNTs were coated with 5 c ALD ZrO_2_ abbreviated as “+5c Zr” as shown in Table 1_._

**Table 1:**
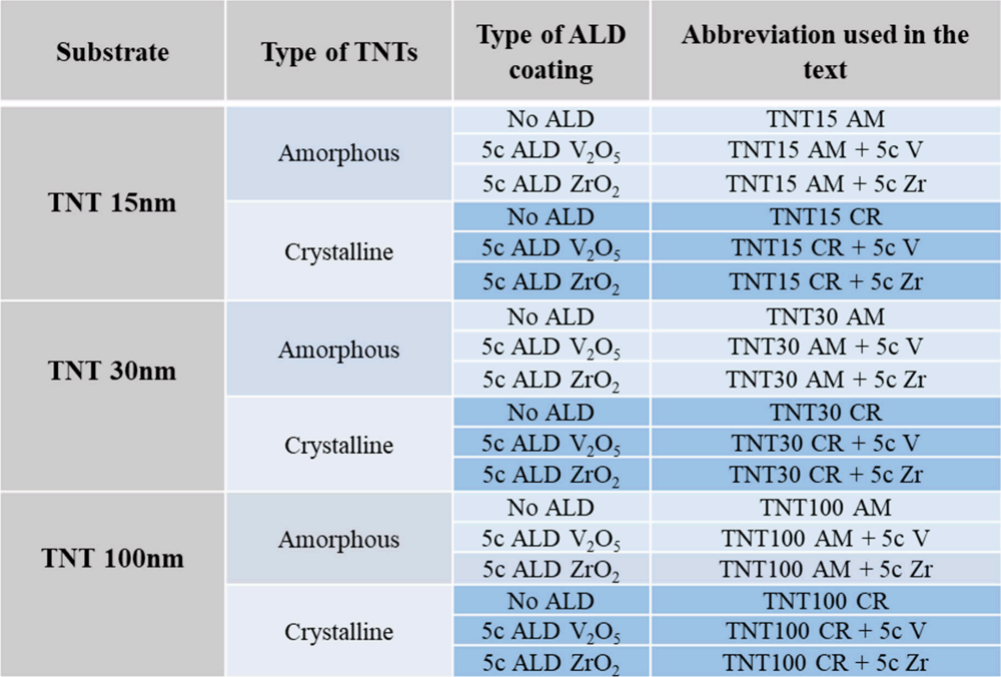
Overview of Different TNTs with ALD Coatings along with Abbreviations Used in This Study

Similarly, ALD of V_2_O_5_ was carried out at 200 °C using tetrakis(dimethylamino)vanadium(IV) (TDMAV) as the vanadium precursor and deionized water as the oxygen source. Under these deposition conditions, one growth ALD V_2_O_5_ cycle (1c) was defined by the following sequence: pulse (500 ms)–N_2_ purge (20 s)–H_2_O pulse (500 ms)–N_2_ purge (20 s). Also in this case, parts of the amorphous and crystalline TNTs were coated with 5c ALD V_2_O_5_ abbreviated as “+5c V” as shown in Table 1_._

The surface morphology of all amorphous and crystalline TNTs was characterized using scanning electron microscopy (SEM, Verios 460 L, FEI). The acquired SEM images were statistically analyzed for morphological parameters using the proprietary Nanomeasure software. The roughness of all TNTs was determined by atomic force microscopy (AFM, Bruker Dimension FastScan) on an area of 5 × 5 cm^2^. Scanasyst-Air tips (*f*_o_ = 70 kHz) were used.

For a detailed characterization of deposited coatings, transmission electron microscopy (TEM, Thermo Fisher Scientific TITAN Themis 60-300) was employed. The TEM was operated at 120 kV in scanning TEM (STEM) mode to collect high-angle annular dark-field (STEM-HAADF) images together with EELS spectrum images by using a Gatan Quantum 966 ERS spectroscope and its annular dark-field detector. The EELS data were collected and processed with SW GMS v3.3, and the presented EELS atomic concentration maps were created with use of a model-based quantification implemented in this SW.

X-ray photoelectron spectroscopy (XPS) was used to examine the surface chemical composition of all amorphous and crystalline 5c ALD ZrO_2_ and 5c ALD V_2_O_5_ coated TNTs. The measurements were conducted with an ESCA 2SR spectrometer from Scienta-Omicron, using a monochromatic Al Kα X-ray source at 250 W, with an energy of 1486.7 eV. The binding energy scale was calibrated against adventitious carbon (284.8 eV), and the analysis was carried out without a charge neutralizer. The collected spectra were processed with CasaXPS software, applying a Shirley-type background correction. Elemental sensitivity factors provided by the equipment manufacturer were used for quantitative analysis.

The water contact angle of the amorphous and crystalline TNTs coated with 5c ALD ZrO_2_ and 5c ALD V_2_O_5_ was assessed using a surface energy evaluation system device (See System E, Advex Instruments, Czech Republic) with proprietary image analysis software. A 3 μL droplet of deionized (DI) water was deposited onto the surface and allowed to stabilize for 5 s. After this time, the contact angle of the droplet was recorded. Measurements were performed 5 times for each type of sample. All results are expressed as the mean ± standard deviation (SD).

### 2.2 Cell Culture

Human osteoblast-like cells MG-63 (ATCC No. CRL-1427; doubling time, DT = 31 h) were cultured in minimum essential medium (Merck) with 10% (v/v) fetal bovine serum (Gibco), 2 mmol L^–1^ glutamine, 1% nonessential amino acids solution, and 50 μg mL^–1^ penicillin/streptomycin solution (Gibco) and maintained at 37 °C in a sterile humidified atmosphere of 5% CO_2_. The cells were proven to be mycoplasma-free, and STR analysis confirmed the origin of the cell line.

### 2.3 Fluorescence Staining and Cell Counting

Before cell cultivation on tested samples, the square-shaped substrates were cut into round shapes with a diameter of approximately 5 mm (using sharp scissors) to fit into the wells used for cell growth. All of the tested materials were sterilized in 70% ethanol for 30 min, washed with deionized water, and dried. The foils were then placed on eight-well chamber slides. Briefly, 200 μL of a suspension of MG-63 cells with a density of 3 × 10^3^ cells/cm^2^ was added to each chamber slide well, seeded, and cultured for 48 h. Cell density was established to maintain optimal cultivation conditions for up to 48 h.

To visualize actin filaments and cell nuclei, phalloidin-FITC and Hoechst 33258 dyes were used, respectively. After being seeded for 48 h, cultured cells were fixed with 3.7% formaldehyde (5 min; 37 °C; dark) and permeabilized with 0.1% Triton X-100 (15 min; 37 °C; dark). Then, 100 μL of phalloidin-FITC (1 μmol L^–1^) was added and the samples were incubated for 40 min at 37 °C. A 10 μL portion of Hoechst 33258 solution was added to the cells 10 min before the end of phalloidin-FITC loading. The final concentration of Hoechst 33258 in a well was 2 μg mL^–1^. The cells were washed twice with phosphate-buffered saline (37 °C). Actin filaments (FITC filter, 480/30 nm) and cell nuclei (DAPI filter, 375/28 nm) were observed with an Eclipse 80i fluorescence microscope (Nikon, Japan). The number of cells grown on the surface was counted from at least 35 fields of view using a NIS-Elements AR instrument (Nikon, Japan). Quantitative analysis of the elongation of cells grown on tested samples was evaluated using a NIS-Elements AR instrument (Nikon, Japan).

### 2.4 Statistics

All biological experiments were repeated at least four times independently. The number of fields was *n* = 35. The number of cell nuclei was related to 1 mm^2^ and expressed as mean ± standard error of the mean (SEM) taken from fluorescence images. Statistical significance was analyzed after normality testing using a one-way ANOVA test followed by a Bonferroni post-test (OriginPro 9.0.0, USA) to compare results to each other at a significance level *p* = 0.05.

## 3 Results and Discussion

### 3.1 Surface, Structure, and Composition

**Figure 1:**
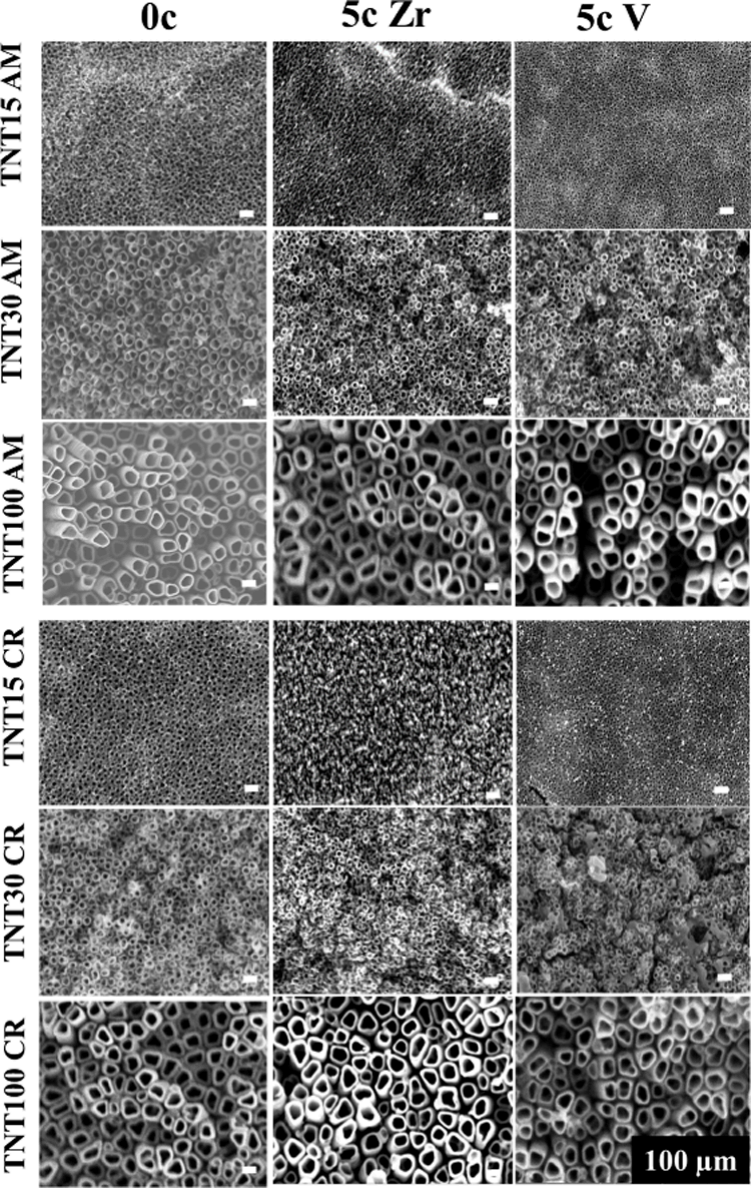
Top-view SEM images of amorphous and crystalline uncoated and coated TNTs with 5c ALD ZrO_2_ and 5c ALD V_2_O_5_.

All amorphous and crystalline TNTs coated with 5c ALD ZrO_2_ and 5c ALD V_2_O_5_ were characterized by using SEM, XPS, and AFM to study their structure, composition, morphology, and surface roughness. Figure 1 shows top-view SEM images of amorphous and crystalline TNTs coated with 5c ALD ZrO_2_ and 5c ALD V_2_O_5_. In our earlier report,^7^ we showed the cross-sectional SEM images of the TNTs, where the same diameters of nanotube layers (also identical anodization conditions) were used. However, from the SEM images, no significant difference can be observed between the uncoated and coated TNTs, as the nominal thickness of the 5c ALD coating is extremely thin (below 1 nm). The nominal thicknesses yielded for 5c ALD ZrO_2_ and 5c ALD V_2_O_5_ are 0.23 and 0.34 nm, respectively, according to the growth rate per ALD cycle, evaluated by variable angle spectroscopic ellipsometry from ZrO_2_ and V_2_O_5_ thin layers deposited on Si wafers. It is important to note that adhesion measurement of the ultrathin films with thickness below 1 nm is currently beyond the instrumental capabilities of existing state-of-the-art tools (including precise nano indenters or AFM-based scratch testers). In addition, due to the nature of ALD film growth (i.e., strong covalent bonds formed with the underlaying substrates), these ALD coatings adhere exceptionally well, compared to any other coatings of similar thickness. Additional inspection of the samples was carried out by STEM-EELS analysis in order to verify the presence of the ALD coatings on the TNTs. Illustrative results from the TNT15 CR+5c V inspection are shown in Figures S1 and S2, showing the coated TNT layers from their top view and side view, respectively. All in all, one can clearly see the presence of V species on all parts of the nanotubes. This confirms the previously demonstrated ability to achieve homogeneous ALD coatings of various compositions on TNTs.^7−12^

**Figure 2:**
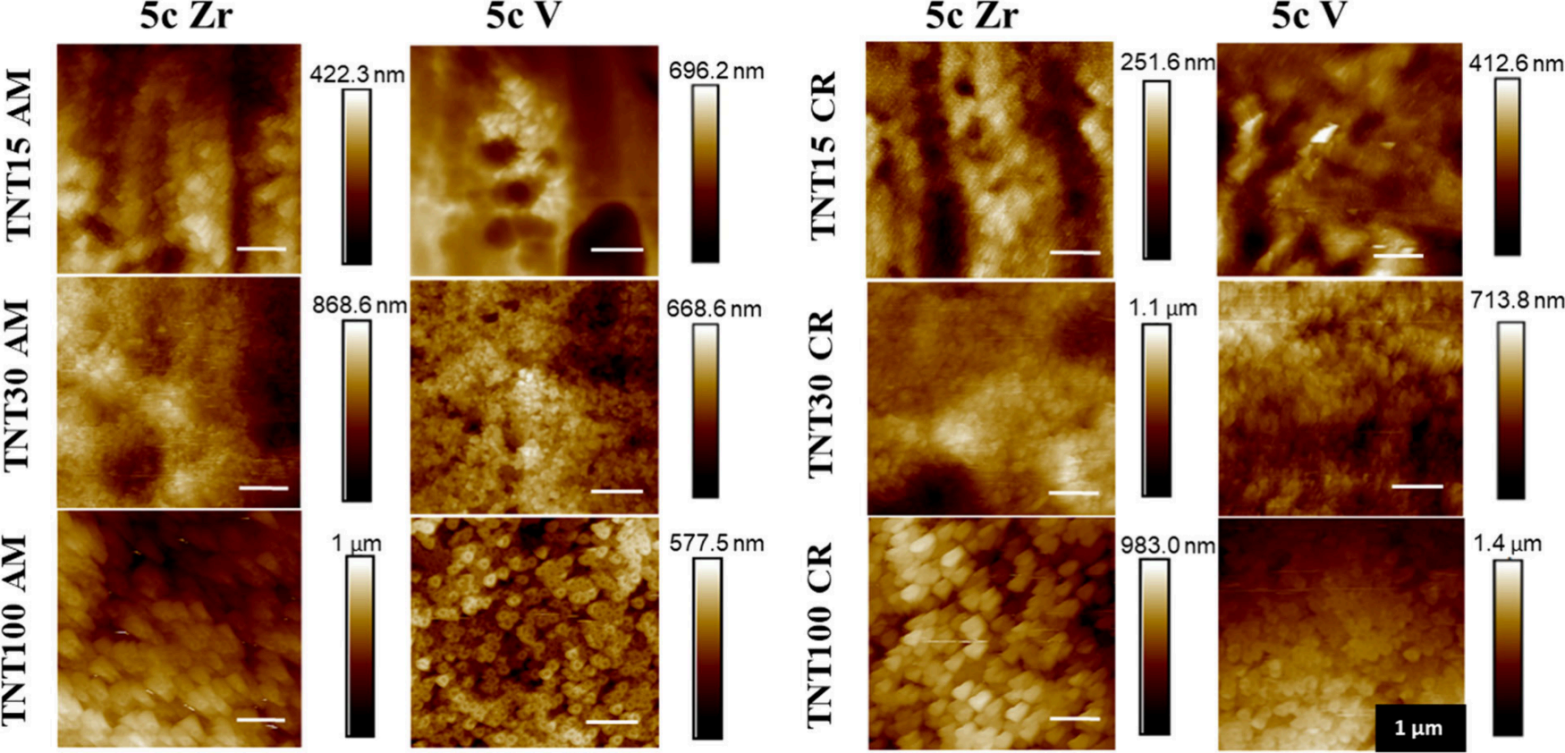
AFM scans across 5 × 5 cm^2^ area of amorphous and crystalline TNTs coated with 5c ALD ZrO_2_ (=5c Zr) and 5c ALD V_2_O_5_ (=5c V).

The roughness of all amorphous and crystalline TNTs coated with 5c ALD ZrO_2_ and 5c ALD V_2_O_5_ was determined by AFM on an area of 5 × 5 cm^2^ as shown in Figure 2. As one can see, nice, sharp AFM images were captured in most of the cases. On the other, one can also see that the topography of the surface is rather rough with features on the submicrometer level. To determine the overall roughness of surfaces, which is the most relevant to describe the cell adhesion on the surface, these images are not the most relevant and are rather illustrative. Thus, the average roughness should be considered instead.

**Figure 3:**
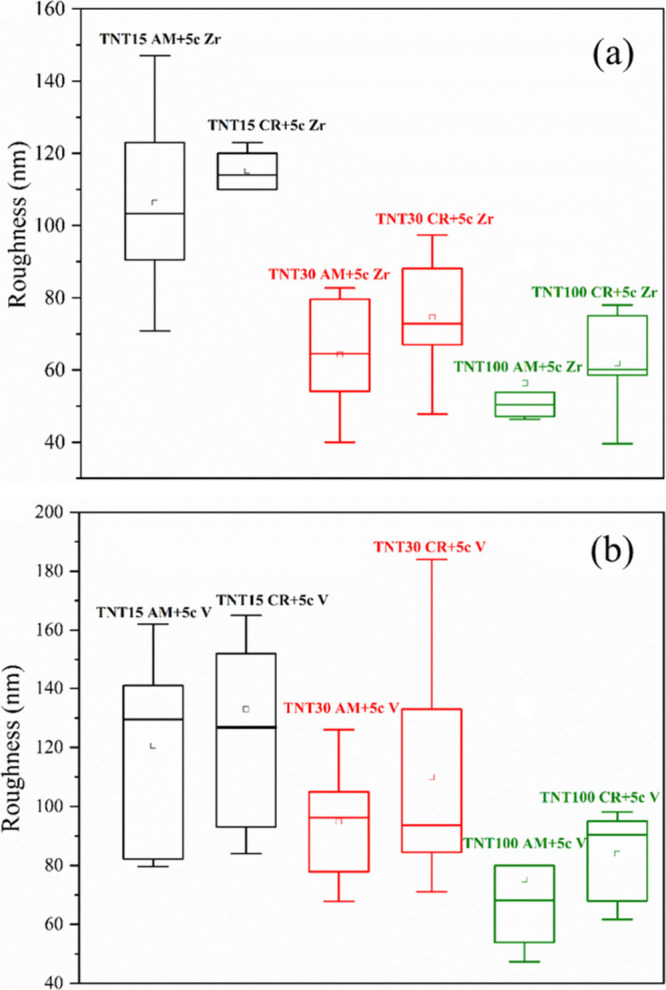
Roughness values (RMS) obtained by AFM in the form of the boxplot describing mean (open square), first and third quantiles (box), and min/max values (whisker) of amorphous and crystalline TNTs coated with (a) 5c ALD ZrO_2_ and (b) 5c ALD V_2_O_5_.

Figure 3 shows the calculated root-mean-square (RMS) values of the surface roughness that are statistically represented as boxplots. The roughness values decrease with the increasing tube diameter of the TNTs, which is in accord with our previous study.^9^ When higher anodization potentials were applied, the surface of TNTs became smoother, as a thicker portion of the initially rough Ti foils was removed through etching, in contrast to a lower voltage. This resulted in noticeable smoothing of the TNT surfaces particularly accentuated with higher potentials. The highest RMS values of approximately 133 nm were obtained for TNT15 CR+5c V followed by approximately 115 nm for TNT15 CR+5c Zr.

**Figure 4:**
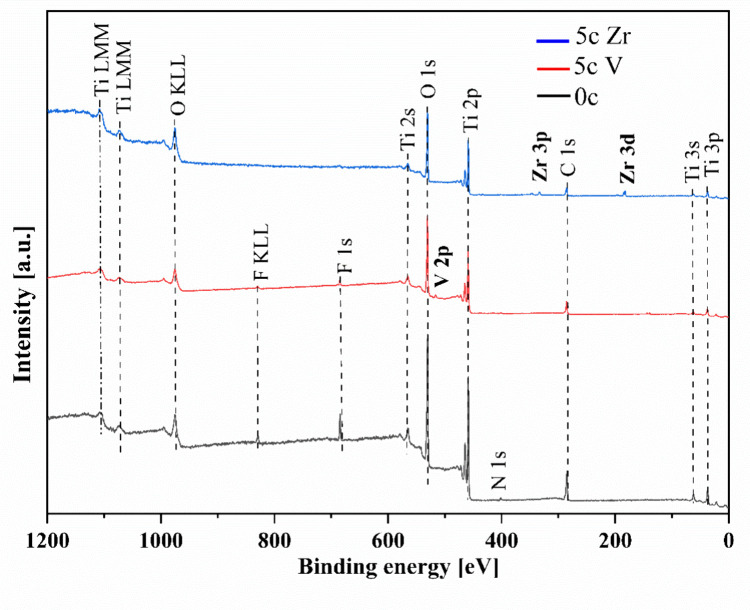
XPS survey spectra of 0c ALD uncoated (black), 5c ALD V_2_O_5_ coated (red), and 5c ALD ZrO_2_ coated (blue) amorphous TNT15.

The XPS spectra were recorded to access the chemical nature of the uncoated (0c ALD), ALD ZrO_2_ coated, and 5c ALD V_2_O_5_ coated TNTs. Figure 4 shows representative XPS surveys for TNT samples with a 15 nm diameter. In all samples, the presence of C, O, N, Ti, and F was detected, as one can see from the atomic percentages (atom %) given in Table 2. The high-resolution XPS spectra acquired in detail (Ti 2p, O 1s, and F 1s) of the uncoated (0c), 5c ALD ZrO_2_, and 5c ALD V_2_O_5_ coated amorphous TNTs are shown in Figure S3. The 3d doublet splitting of 2.38 eV between Zr 3d_3/2_ (184.78 eV) and Zr 3d_5/2_ (BE = 182.4 eV) appears on the high resolution XPS spectra, which corresponds to ZrO_2_,^28^ as shown in Figure S3a. Similarly, for the V_2_O_5_ coated TNTs, the V 2p_3/2_ and V 2p_1/2_ peaks were found at 517.1 and 524.7 eV, respectively, confirming the formation of the V_2_O_5_ phase (V^5+^),^29^ as shown in Figure S3b. The presence of O, Zr, and V (in atom %) and the ratio of Zr:O and V:O in TNT15 AM+5c Zr and TNT15 AM+5c V respectively are shown in Table S1.

**Table 2:** Atomic Concentration (%) of 0c ALD Coated, 5c ALD ZrO_2_ Coated, and 5c ALD V_2_O_5_ Coated Amorphous TNT15 Derived from the XPS Spectra

	atomic concentration (%)						
	C	O	Zr	N	V	F	Ti
0c	27.47	47.25		1.50		6.35	17.43
5c Zr	23.30	53.87	2.09			2.30	18.43
5c V	10.55	53.64			2.69	3.32	29.81

The wettability of all samples investigated in this study (as given in Table 1) was characterized by contact angle measurements under equilibrium conditions (i.e., after 1 week of relaxation on the air and under ambient light conditions, which is the same status as for the samples used for the cell test).

**Figure 5:**
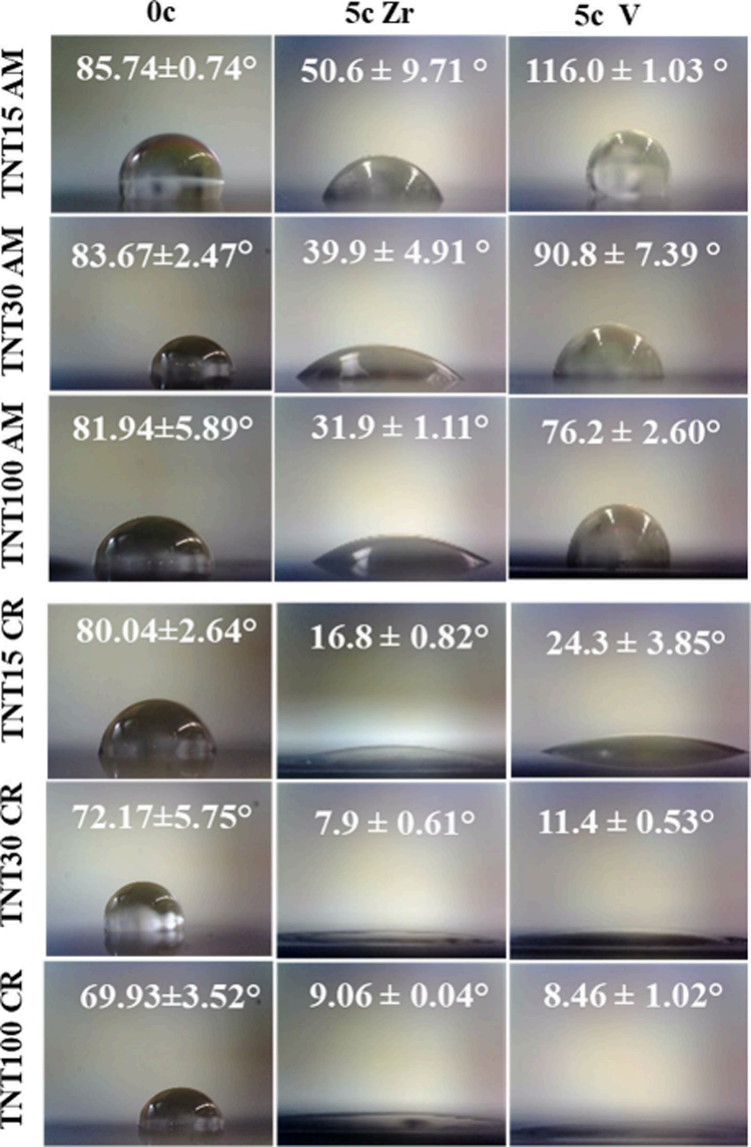
Contact angle measurements of amorphous and crystalline uncoated TNTs and TNTs coated with 5c ALD ZrO_2_ and 5c ALD V_2_O_5_. Each image shows a 3 μL droplet on the surface. The white digits give the statistical mean and SD of the contact angles.

Figure 5 shows photographs of droplets on all samples, and it gives corresponding measured water contact angles (WCAs). There results clearly confirm the hydrophilicity of the TNTs themselves (please see the left column for 0c). Upon closer look, one sees differences in WCA among TNTs with different diameters. As established in our previous study,^9^ the WCA of uncoated (as well as TNTs coated with 5c ALD TiO_2_) decreased with an increasing tube diameter. We observed the strong dependence of the WCA on the tube diameters irrespective of the ultrathin TiO_2_ coating. These results are in accord with the classical Wenzel model,^30^ where a droplet fills up a rough surface and forms fully wet contact depending on the roughness factor and the surface free energy. Hence, when a high surface energy material is combined with micro- and nanoscale roughness, it results in the formation of a superhydrophilic surface.^31^ Larger tube diameters provide a more accessible pathway for the liquid to penetrate within nanotubes, thus yielding the lower contact angle.

However, additional ALD coatings on the surfaces (see middle and right columns of Figure 5) make the situation more complex. An effect of the additional 5c ALD ZrO_2_ and 5c ALD V_2_O_5_ coatings on TNTs on lowering of the WCA compared to uncoated TNTs is clearly present. What is more, there is a clear difference between ALD coated samples that have an amorphous and a crystalline TNT structure. Let us discuss first the amorphous samples. The amorphous TNTs with a 5c ALD ZrO_2_ coating make the surfaces significantly more hydrophilic compared to the uncoated TNTs. WCA of TNT15AM decreased from 85.74 ± 0.74° to 50.6 ± 9.71° for TNT15AM+5c Zr. On the other hand, TNT15AM+5c V shows a hydrophobic nature with a WCA of 116.0 ± 1.03°. This hydrophobic state of TNT15AM+5c V and TNT30AM+5c V can be due to the intercalated alkyl chains as observed in nanostructured V_2_O_5_.^32^ In contrast, TNT100AM+5c V shows a comparably stronger hydrophilic nature (76.2 ± 2.60°) due to the significantly larger tube diameters compared to TNT15AM+5c V and TNT30AM+5c V and this parameter seems thus to prevail.

However, on all of the annealed 5c ALD ZrO_2_ and 5c ALD V_2_O_5_ TNTs, the water droplet spreads entirely. Annealed TNTs are on average rougher than amorphous counterparts, as shown in Figure 3. This can be attributed to the exposure to increased temperatures (during annealing of the TNTs themselves), which results in larger TiO_2_ grain size and higher roughness. However, the additional 5c ALD ZrO_2_ and 5c ALD V_2_O_5_ make on average all these coated TNTs even slightly rougher for currently unknown reasons. At the same time, the chemistry of the surfaces is changed, as they are coated with different oxides (as shown by XPS and TEM results). All together, these factors have a tremendous effect on lowering the WCA to the superhydrophilic level.

In an effort to link our results to the findings described in the literature, an extensive literature survey was carried out. It turned out that comparative studies on the wetting of different metal oxides on different substrates are scarce in the literature. In fact, only one paper discusses comparison of WCA of TiO_2_ and ZrO_2_, and it shows that ZrO_2_ tends to be more wetting compared to TiO_2_.^33^ So this would sort of explain the trend for our ZrO_2_ films. Nevertheless, the reason for the superhydrophilicity of the 5c ALD V_2_O_5_ coated annealed TNTs is unknown. A detailed XPS investigation of V peaks on annealed and amorphous TNTs did not show any difference (data not shown).

Based on findings presented in this work, it can be assumed that the superhydrophilic nature of annealed TNTs with ALD coatings is based on the combined effect of the roughness and chemical composition that play a joint role by comparing the magnitude of changes in roughness (Figure 3) and WCA (Figure 5) upon ALD coatings; it appears though that the increased roughness of the annealed TNTs (compared to amorphous ones) and also of the annealed coated TNTs (compared to uncoated ones) prevails in its effect over the effect of the chemical nature of the coatings composed of different oxides.

To shed more light on this interplay, a side set of wetting experiments was carried out on planar Ti foils (i.e., without nanotubular layers) in the uncoated state and ALD coated with 5c of TiO_2_, ZrO_2_, and V_2_O_5_.

Interestingly, the influence of all of these oxides on the WCA is completely different on Ti foils, as shown Figure S4. In other words, the difference in WCA for uncoated and coated flat Ti foils is not very pronounced. All coatings tend to keep the hydrophilic nature of the surface. Apparently, the nanotube morphology is a game changer. A deeper investigation will be carried out on these interesting aspects in the future.

### 3.2 Cell Growth on Tested Surfaces

This study focuses on estimating the effect of amorphous or crystalline TNT layers coated by ZrO_2_ and V_2_O_5_ ALD on the growth of osteoblast-like MG-63 cells. In the context of number of cycles, it follows our previous reports showing the beneficial effect of 5c TiO_2_-coated surfaces.^7,9^ In this case, additional 5c ZrO_2_ and 5c V_2_O_5_ ALD coatings were applied on the amorphous and crystalline nanotube layers with 15, 30, and 100 nm diameters, with the aim of studying their effect on cell adhesion, proliferation, and growth.

**Figure 6:**
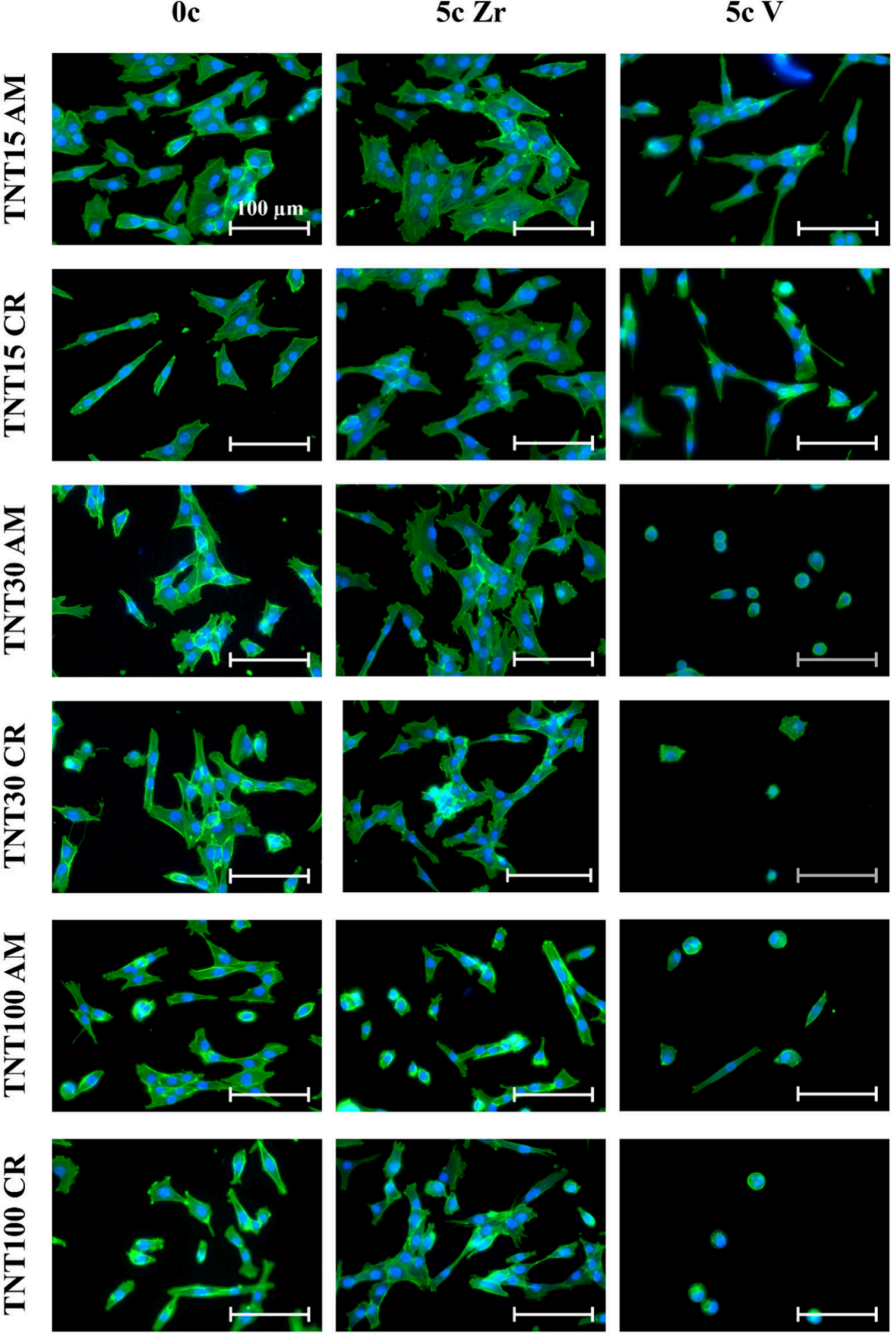
Photomicrographs of MG-63 cells grown on uncoated or 5c ZrO_2_ or 5c V_2_O_5_ ALD-coated amorphous and crystalline TNT15, TNT30, and TNT100 for 48 h (0c = without ALD coating; 5c Zr = 5c ZrO_2_ ALD coating; 5c V = 5c V_2_O_5_ ALD coating). The actin filaments were stained with the Phalloidin-FITC probe (green), and the cell′s nuclei were stained with the Hoechst 33258 probe (blue).

MG-63 cells were cultured on the tested samples for 48 h. This incubation period is sufficient for evaluating the cell adhesion, elongation, proliferation, and morphology on all of the investigated surfaces. The average time for cell attachment to plastic surfaces under standard culture conditions is generally around 6 h, with many cell lines fully adhering within 24 h.^34,35^ The doubling time of MG-63 cells was approximately 31 h, so we characterized both cell adhesion and cell proliferation on the tested samples. Based on the literature, fluorescence staining of actin filaments and cell nuclei was utilized to describe the functional morphology of MG-63 cells.^36,37^ Photomicrographs of cells grown on uncoated or ALD ZrO_2_ and V_2_O_5_ ALD coated TNT layers are shown in Figure 6. As one can see, both ultrathin coatings have diverse effects on the cell morphology.

**Figure 7:**
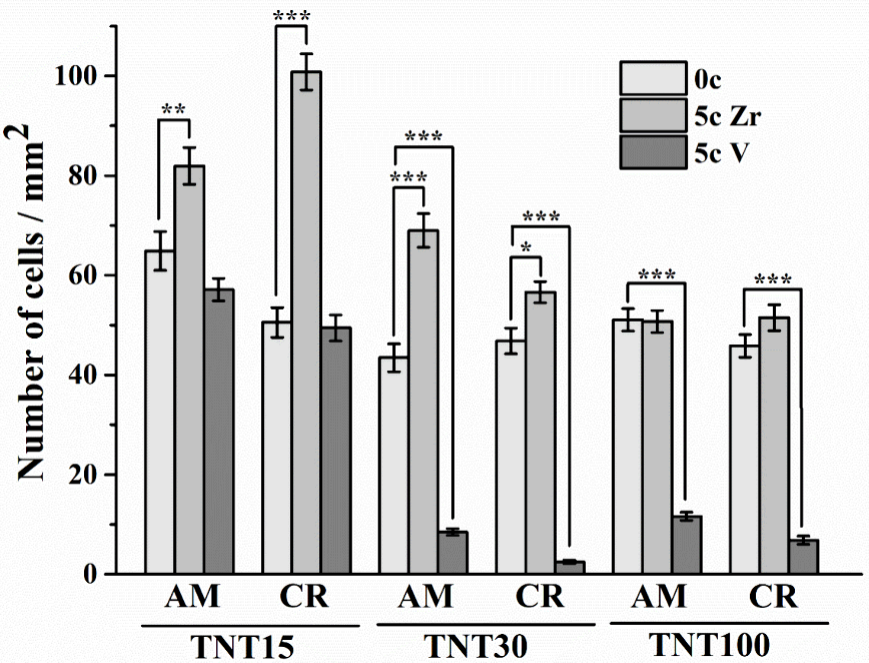
Density of MG-63 cells grown on uncoated or 5c ZrO_2_ and 5c V_2_O_5_ ALD-coated amorphous and crystalline TNT15, TNT30, and TNT100 for 48 h (0c = without ALD coating; 5c Zr = 5c ZrO_2_ ALD coating; 5c V = 5c V_2_O_5_ ALD coating). Data originated from four independent experiments presented as mean ± SEM (*, *p* < 0.05; **, *p* < 0.01; ***, *p* < 0.001).

The images from fluorescence staining were subjected to image analysis to quantify the cell density. The cell nuclei were counted in individual fields of view and related to an area (mm^2^) according to previous reports.^8,38−40^ The results on densities of MG-63 cells grown on ZrO_2_ and V_2_O_5_ ALD-coated amorphous or crystalline TNT layers compared to uncoated samples are shown in Figure 7. We found that only 5c ZrO_2_ ALD coatings significantly increased the cell density on both amorphous and crystalline TNT15 and TNT30, compared with uncoated TNT15 and TNT30 after 48 h.

It has been shown that the chemical composition, topography of surfaces, and crystal structure of TNT layers play crucial roles in cell adhesion, proliferation, osseointegration, and mineralization.^41,42^ In a recent study, 350 cycles of ZrO_2_ ALD coatings on pure Ti improved the biocompatibility of osteoblasts and fibroblasts.^43^ Similarly, hundreds of ZrO_2_ ALD cycles increased the adhesion and viability of MC3T3-E1 cells on Mg–Sr alloy^44^ and Zn–Li alloy.^45^ In contrast, the density of MG-63 cells grown on 5c V_2_O_5_ ALD TNT15 was comparable to that on uncoated TNT15. Interestingly, the most beneficial effect on cell growth was found in 5c ZrO_2_ ALD TNT15 CR. For these particular samples, the cell density was approximately two times higher compared to uncoated TNT15 CR, demonstrating the excellent biocompatibility of 5c ZrO_2_ ALD TNT15 CR. This was most likely caused by the changes in the surface chemistry of ALD-coated surfaces.

In terms of surface chemistry, ZrO_2_ is generally considered to be a viable and cell-compatible material.^46,47^ Thus, an ultrathin ZrO_2_ coating achieved by 5 cycles of ZrO_2_ ALD partially shaded the impurities of F-based species in tested TNT layers, while still preserving the original ultrathin structure and morphology. Similarly, the concentration of F-species decreased in the case of the 5c ALD V_2_O_5_ coating, as demonstrated by XPS results in Table 2. On the other hand, an adverse effect of V-based species on cell growth was deepened after modification of TNTs by 5c ALD V_2_O_5_ compared to uncoated TNTs, as mentioned above. Therefore, the beneficial effect of ZrO_2_ ALD is 2-fold: (i) it shades impurity elements on TNTs leading to biocompatibility improvement, also mentioned in our previous studies,^8,10^ and (ii) being very biocompatible with cells, it further increases their adhesion, proliferation, and osseointegration.

Another important factor that may influence cell growth on ALD-coated surfaces is the crystallinity of the TNTs. The proliferation and mineralization activities of osteoblastic cells were increased after cultivation on anatase or a mixture of anatase and rutile TNT layers compared to amorphous samples.^48^ The increase in activity of osteoblasts grown on TNT surfaces with a rutile crystal structure can be attributed to the higher adsorption of proteins to the surfaces.^41^ Next, the crystalline TNT15 coated by ALD showed the highest roughness values. Similarly, the higher roughness of Ti-6Al-4V alloy with 100c hydroxyapatite-ALD coating was optimal for fibroblast adhesion and proliferation, compared to smoother surfaces.^49^ Moreover, the roughness of surfaces decreased with the increasing diameter of the TNTs.^9^

According to the results on characterization of cell growth on TNT30 and TNT100 coated by 5c V_2_O_5_ ALD, the finding of low cell densities on these surfaces was attributed to the adverse effect of V species. In previous studies, the authors described that the decreased cell growth on V-coated surfaces can be explained by the toxic effect of V oxides and V ionic species on the layers or their release from these surfaces into the surroundings.^13,50,51^ Nevertheless, there has been no study that would assess the effects of V oxide ALD on cell adhesion and growth until this work. In the case of 5c ZrO_2_ ALD TNT100, the cell growth on uncoated TNT100 was comparable to that on 5c ZrO_2_ ALD surfaces. It seems that the comparability of cell growth between uncoated TNT 100 and 5c ZrO_2_ ALD TNT100 in our results is primarily influenced by TNT diameter, according to the outcomes of previous reports.^52,53^

The significance of hydrophilicity/hydrophobicity on the cell adhesion and the cell growth on different modified surfaces was reported.^54,55^ Generally, optimal surface hydrophilicity improves protein adsorption, cell adhesion, and proliferation immediately after seeding. In contrast, more hydrophobic surfaces fail to maintain consistent contact with cells, limiting their adhesion potential.^56,57^

The results of the WCA measurements in Figure 5 showed that ALD coatings changed the surface wettability significantly. After the deposition of 5c ZrO_2_ ALD on TNT AM, the water wettability was enhanced, and thus, the hydrophilicity was increased. These findings were supported by the beneficial effect of the 5c ZrO_2_ ALD coating on the cell growth provided in Figure 6. In 5c ZrO_2_ and V_2_O_5_ ALD TNT CR, the contact angle was in the range of 8–24°, indicating that it is highly hydrophilic. Given this low contact angle of under 10°, the TNT surfaces can be classified as near superhydrophilic.

Interestingly, 5c V_2_O_5_ ALD TNT15 AM surfaces exhibited more hydrophobic properties compared to uncoated TNT15 AM. There is no study that would describe the effects of surface wettability of ALD ZrO_2_ and 5c ALD V_2_O_5_ coated samples on cell adhesion and proliferation until this work.

### 3.3 Cell Elongation

**Figure 8:**
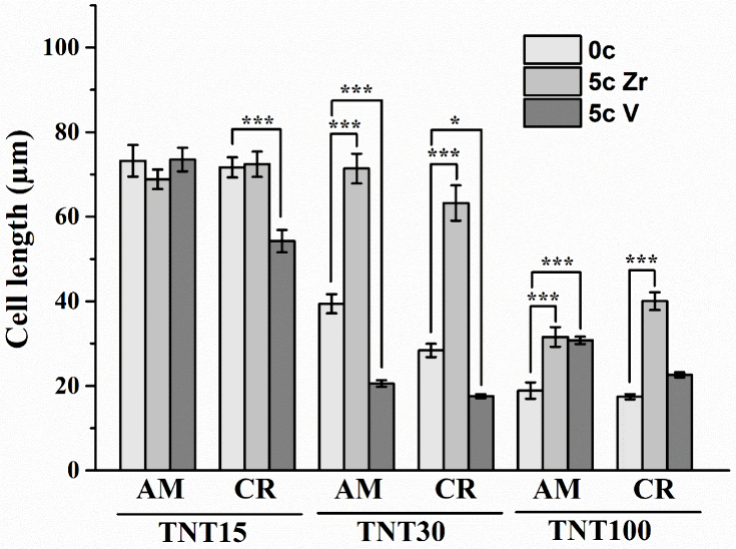
Analysis of elongation of MG-63 cells grown on uncoated or 5c ZrO_2_ and 5c V_2_O_5_ ALD-coated amorphous and crystalline TNT15, TNT30, and TNT100 grown for 48 h (0c = without ALD coating; 5c Zr = 5c ZrO_2_ ALD coating; 5c V = 5c V_2_O_5_ ALD coating). Data originated from four independent experiments presented as mean ± SEM (*, *p* < 0.05; **, *p* < 0.01; ***, *p* < 0.001).

To evaluate the cell interaction with amorphous and crystalline ALD ZrO_2_ and 5c ALD V_2_O_5_ TNT layers, the elongation of MG-63 cells after 48 h was evaluated after the fluorescence staining. The occurrence of elongated MG-63 cells reflects the proper attachment and adhesion of cells to the surfaces. The surface topography not only induces cellular orientation but also supports elongation along the direction of the grooves.^58,59^ Assessment of elongated MG-63 cells on uncoated or ALD-coated TNT layers is provided in Figure 8.

There was no difference in lengths among cells grown on uncoated or 5c ZrO_2_ ALD coated TNT15 AM or TNT15 CR for 48 h. However, the amorphous and crystalline 5c ZrO_2_ ALD TNT15 caused MG-63 cells to exhibit a well-developed cytoskeleton compared to those grown on uncoated TNT15 (Figure 5). A decrease in elongation of MG-63 cells by approximately 20% was observed on 5c V_2_O_5_ ALD TNT15 CR compared with that of uncoated ones.

The 5c ZrO_2_ ALD coating influenced the elongation of MG-63 cells cultured on TNT30 AM and TNT30 CR, and the cells reached a double length compared to those grown on uncoated TNT30. The morphology of MG-63 cells grown on 5c ZrO_2_ ALD TNT30 was analogous to those on 5c ZrO_2_ ALD TNT15. The elongated morphology of MG-63 cells on ALD-coated surfaces was found also in our recent study.^8^ Another report presented that, after 4000c hydroxyapatite ALD coating, MC3T3-E1 cells were spread uniformly and morphology was more elongated after 48 h. On the cover glass, cell morphology was relatively circular.^36^ On the other hand, the morphology of L928 and MC3T3-E1 cells on surfaces coated by 350c ZrO_2_ ALD was analogous to those on Ti discs after 3 and 4 days of cultivation.^43^

The elongation in cells grown on TNT30 AM and TNT30 CR coated by 5c V_2_O_5_ ALD decreased significantly. The cells grown on 5c V_2_O_5_ ALD TNT30 and TNT 100 exhibited a circle-shaped morphology after 48 h, as shown in Figure 5. The length of MG-63 cells cultured on the amorphous and crystalline 5c ZrO_2_ ALD TNT100 and amorphous 5c V_2_O_5_ ALD TNT100 was increased compared to that on uncoated TNT100.

## Conclusions

For the first time, the growth of osteoblastic MG-63 cells was evaluated on surfaces of amorphous/crystalline TNT layers coated with ultrathin ZrO_2_ and V_2_O_5_ coatings produced by ALD in comparison to uncoated ones. We estimated the coated surfaces in terms of the effect of tube diameter, crystallinity, roughness, wettability, and surface chemistry on cell growth and elongation. We showed that crystalline 5c ZrO_2_ TNT15 exhibited the most favorable effect on the cell growth and cytoskeleton development after 48 h of incubation. In contrast, the cell growth on 5c V_2_O_5_ ALD TNT15 was comparable to that on uncoated TNT15. With the increasing nanotube diameter, there were decreases in the cell density and elongation according to the results from testing of both AM and CR TNT30 or TNT100. In parallel, an overall decrease of surface roughness was observed with increasing nanotube diameter and added coatings. The expected cytotoxic effect of 5c V_2_O_5_ ALD TNT30 and TNT100 on MG-63 cells was confirmed. Our findings suggest that ultrathin ZrO_2_ ALD coatings hold significant promise for enhancing the biocompatibility of various surfaces, demonstrating strong potential for use in biomedical applications.

## Supporting Information Available

High-angle annular dark-field STEM-EELS images of the coated TNTs with 5c ALD ZrO_2_ and 5c ALD V_2_O_5_, XPS spectra and composition of amorphous TNT15 with 5c ALD ZrO_2_ and 5c ALD V_2_O_5_, and water contact angle measurement of Ti foils with No ALD, 5c ALD ZrO_2_, and 5c ALD V_2_O_5_ (PDF)
