# Supplementary material for: Ultrathin ALD Coatings of Zr and V Oxides on Anodic TiO2 Nanotube Layers: Comparison of the Osteoblast Cell Growth
Source: ACS Appl Mater Interfaces. 2024 Dec 28;17(1):739–49. doi: 10.1021/acsami.4c19142 (PMC11783542; doi:10.1021/acsami.4c19142)
Supplement: Supplementary file 1 — am4c19142_si_001.pdf [file am4c19142_si_001.pdf]

Supporting Information

Ultrathin ALD Coatings of Zr and V Oxides on Anodic TiO_2 Nanotube Layers: Comparison of the Osteoblast Cell growth

Kaushik Baishya[a,1], Jana Bacova[b,1], Bachar Al Chimali[c], Jan Capek[b], Jan Michalicka[a], Gael Gautier [c], Brice Le Borgne [c], Tomas Rousar [b*], Jan M. Macak [a,d*]

[a] *Central European Institute of Technology, Brno University of Technology, Purkynova 123, 612 00 Brno, Czech Republic.*

[b] *Department of Biological and Biochemical Sciences, Faculty of Chemical Technology, University of Pardubice, Studentská 573, 53210 Pardubice, Czech Republic*

[c] *GREMAN UMR-CNRS 7347, INSA Centre Val de Loire, Université de Tours, 37071 Tours Cedex 2, France*

[d] *Center of Materials and Nanotechnologies, Faculty of Chemical Technology, University of Pardubice, Nam. Cs. Legii, 532 10 Pardubice, Czech Republic.*

** Corresponding authors:*

Dr. Jan M. Macak, Center of Materials and Nanotechnologies, Faculty of Chemical Technology, University of Pardubice, Nam. Cs. Legii, 532 10 Pardubice, Czech Republic.

E-mail: jan.macak@upce.cz

Prof. Tomas Rousar, Department of Biological and Biochemical Sciences, Faculty of Chemical Technology, University of Pardubice, Studentska 573, 53210 Pardubice, Czech Republic

E-mail: tomas.rousar@upce.cz

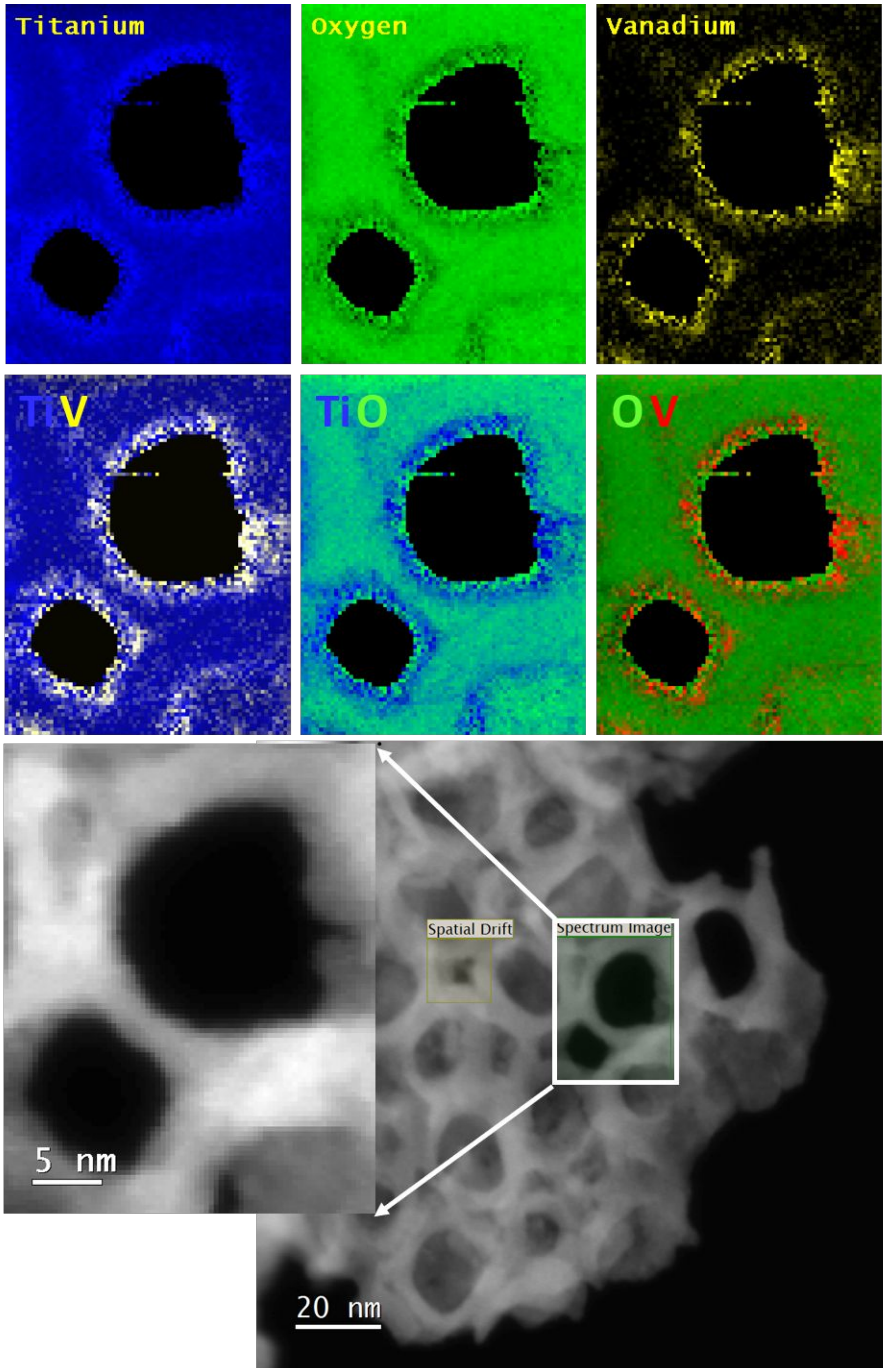

Figure S1. STEM-EELS analysis of the coated nanotubes in a top-view. The results include a STEM-HAADF image (in the bottom in grey scale) and corresponding EELS maps of Ti-L, O-K and V-L edges and their overlays.

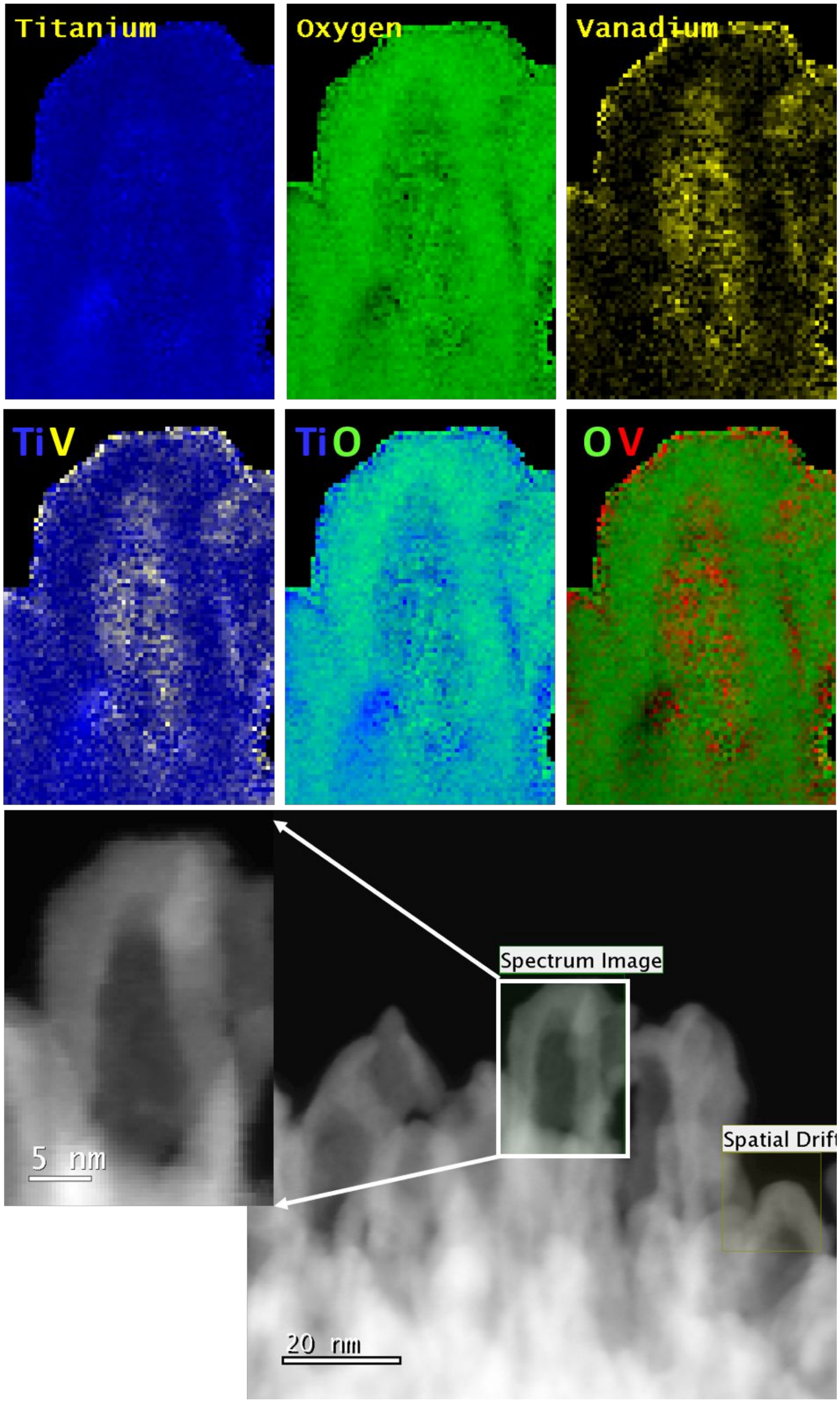

Figure S2. STEM-EELS analysis of the coated nanotubes in a side-view. The results include a STEM-HAADF image (in the bottom in grey scale) and corresponding EELS maps of Ti-L, O-K and V-L edges and their overlays.

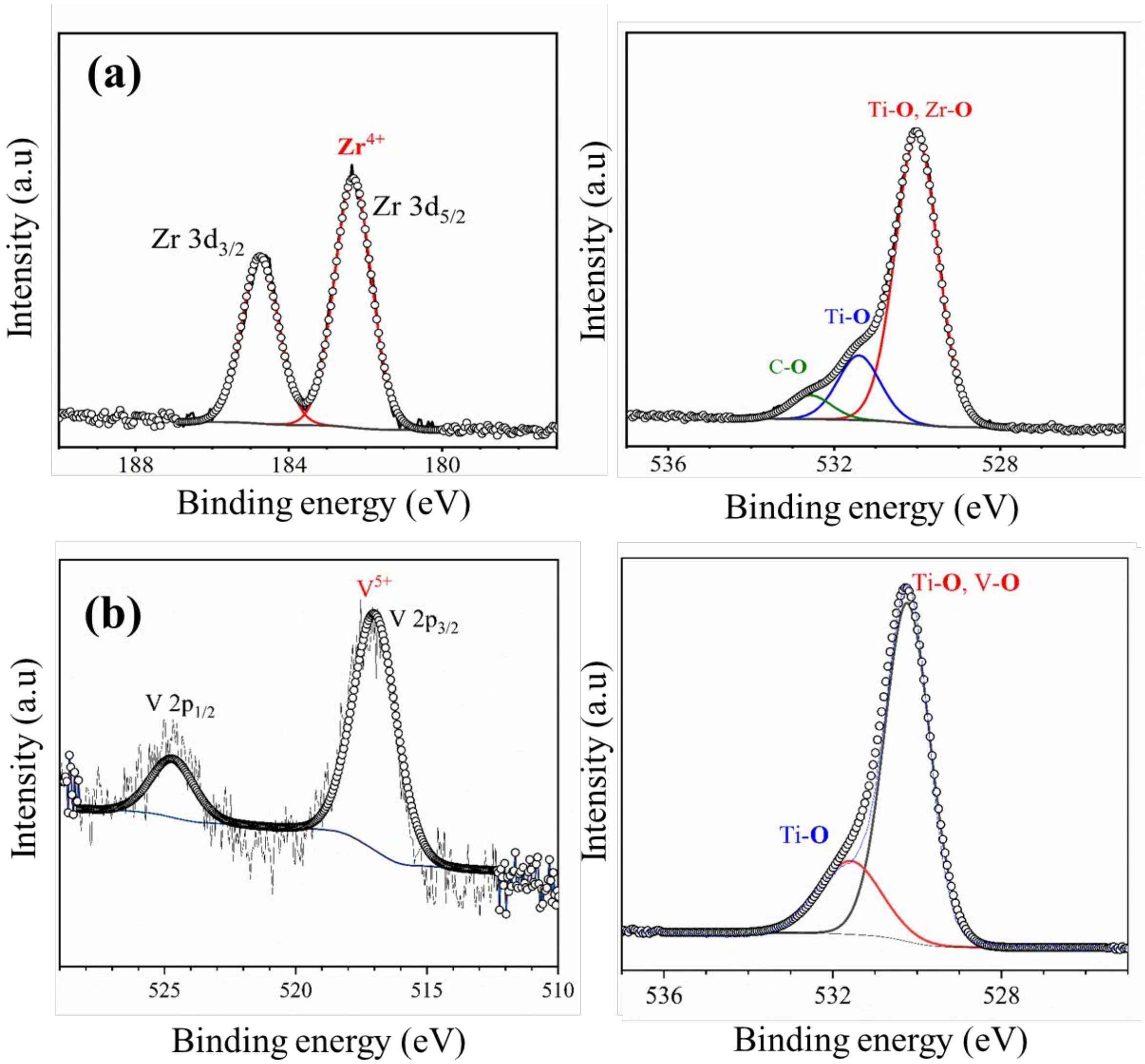

Figure S3. High resolution XPS spectra of (a) TNT15 AM+5c Zr and (b) TNT15 AM+5c V.

Table S1. XPS chemical composition (atomic %) of TNT15 AM+5c Zr and TNT15 AM+5c V.

	**O**	**Zr**	**V**	**V:O**	**Zr:O**
**5c Zr**	67	29	-	-	0.43
**5c V**	65.3	-	31.2	0.47	-

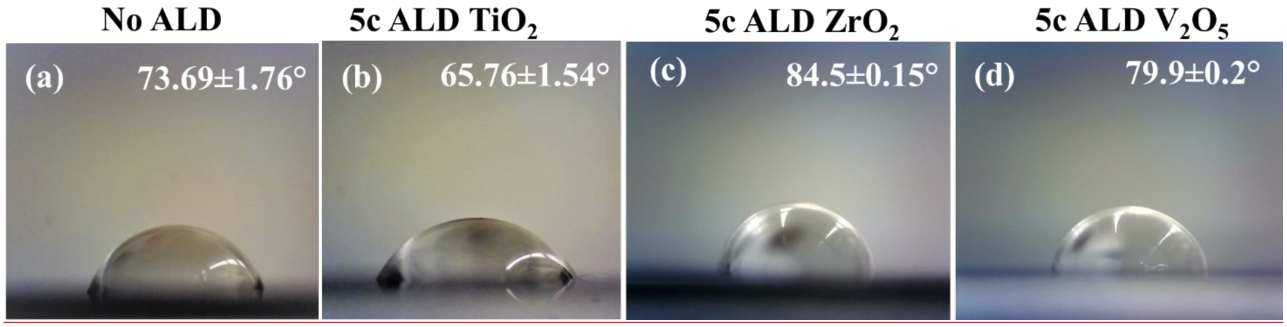

Figure S4. The contact angle measurements of Ti foils with (a) no ALD (b) 5c ALD TiO_2 (c) 5c ALD ZrO_2 and (d) 5c ALD V_2O_5. Each image shows a 3 μL droplet on the surface. The white digits give the statistical mean and SD of contact angles.
